# Chitosan Edible Films and Coatings with Added Bioactive Compounds: Antibacterial and Antioxidant Properties and Their Application to Food Products: A Review

**DOI:** 10.3390/polym15020396

**Published:** 2023-01-12

**Authors:** Nuria Muñoz-Tebar, José A. Pérez-Álvarez, Juana Fernández-López, Manuel Viuda-Martos

**Affiliations:** IPOA Research Group, Agro-Food Technology Department, Centro de Investigación e Innovación Agroalimentaria y Agroambiental (CIAGRO-UMH), Miguel Hernández University, 03312 Orihuela, Spain

**Keywords:** chitosan, edible films, meat, dairy, fruits, antimicrobial, antioxidant

## Abstract

Chitosan is the deacetylated form of chitin regarded as one of the most abundant polymers and due to its properties, both chitosan alone or in combination with bioactive substances for the production of biodegradable films and coatings is gaining attention in terms of applications in the food industry. To enhance the antimicrobial and antioxidant properties of chitosan, a vast variety of plant extracts have been incorporated to meet consumer demands for more environmentally friendly and synthetic preservative-free foods. This review provides knowledge about the antioxidant and antibacterial properties of chitosan films and coatings enriched with natural extracts as well as their applications in various food products and the effects they had on them. In a nutshell, it has been demonstrated that chitosan can act as a coating or packaging material with excellent antimicrobial and antioxidant properties in addition to its biodegradability, biocompatibility, and non-toxicity. However, further research should be carried out to widen the applications of bioactive chitosan coatings to more foods and industries as well was their industrial scale-up, thus helping to minimize the use of plastic materials.

## 1. Introduction

The main function of food packaging is to isolate food product(s) from the surrounding environment, diminishing or inhibiting contact with spoilage factors such as microorganisms, oxygen, temperature, and/or moisture to avoid or delay loss of nutritional value or quality and consequently improve and extend the product’s shelf life [1]. For this reason, as well as for convenience and communication with consumers, the food industry is the main user of packaging materials. However, the growing concern about the environmental impact generated by these activities has meant a turning point in the search for biodegradable and more eco-friendly alternatives [2,3]. In this sense, there are several compounds that could be used as biodegradable materials to manufacture food packaging such as films or coatings. To elaborate, biodegradable films and several coating materials can be used including polysaccharides (starch, cellulose, pectin, gums, or chitosan); lipids such as waxes, oils, and fats, and animal proteins (casein, gelatin, or whey proteins); and vegetal proteins (zein, soy proteins, or gluten) or polysaccharides produced by microorganisms (xanthan gum or pullulan).

**Figure 1:**
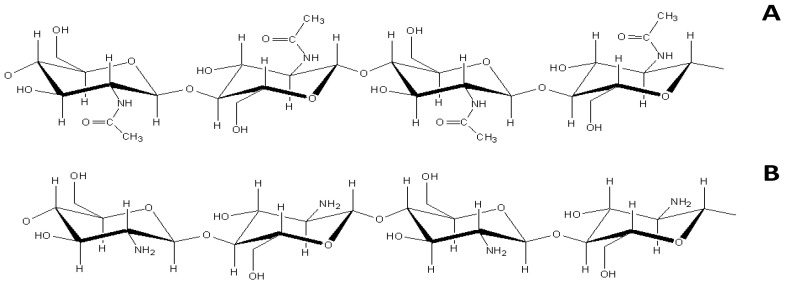
(**A**) Chemical structure of chitin; (**B**) Chemical structure of chitosan.

Nowadays, chitosan is one of the best-known and most widely used polysaccharides in preparing edible films and coatings due to its high antimicrobial activity, biocompatibility, biodegradability, and non-toxic profile [4]. Chitosan is a polysaccharide of N-acetyl D-glucosamine and D-glucosamine units and is mainly obtained by the partial deacetylation of chitin (Figure 1) [5]. Chitin is known as a crucial structural polymer that constitutes a big portion of insect and crustacean exoskeletons.

**Figure 2:**
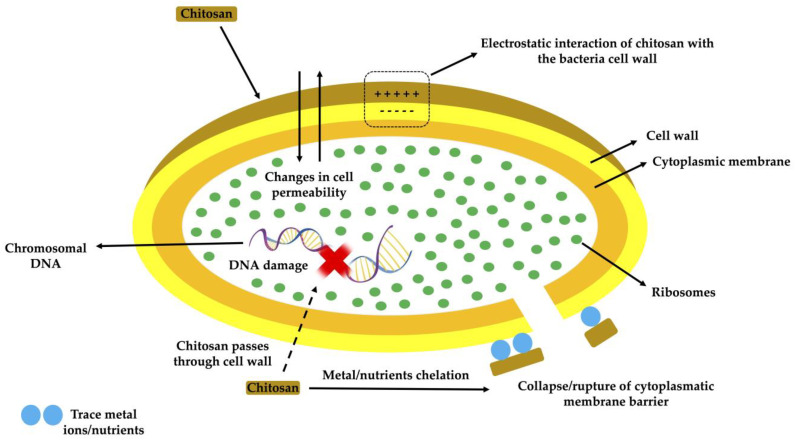
Schematic illustration of antimicrobial mechanisms of chitosan.

Among the various properties, it is important to highlight that chitosan is a natural cationic polymer, while the majority of polysaccharides are either neutral or have an anionic charge. This property permits the production of multilayer structures or electrostatic complexes with other synthetic polymers or negatively charged natural ones [4]. Additionally, chitosan has the property of forming films. For the formulation of edible chitosan films and coatings, the concentrations vary from 1 to 3% (*w*/*v*) and are dissolved in an aqueous solution acidified with acetic acid or lactic acid in concentrations that ranged between 1% and 3% (*v*/*v*) [6]. Edible films and coatings obtained from chitosan are characterized by being transparent or slightly yellowish, with a smooth surface, flexible and cohesive, with a highly mechanical resistance (comparable to the many commercial polymers), hydrophilic, innocuous, biocompatible, biodegradable, and suitable for a wide range of food groups [7,8,9]. Finally, another very important property of chitosan is its strong antimicrobial activity against Gram-positive and Gram-negative bacteria in addition to fungi [10]. These antimicrobial properties depend on several factors including pathogen type, pH of the media, structural properties (such as deacetylation degree and molecular weight) source, as well as the concentration of chitosan [9]. Several action mechanisms, as can be seen in Figure 2, have been proposed to explain the antimicrobial activity of chitosan

The properties of chitosan edible films and coatings can vary depending on the degree of deacetylation and molecular weight of the chitosan used in its formulation, plasticizers, and viscosity among others. Depending on these factors, the gas permeability to oxygen and carbon dioxide as well as the mechanical and thermal properties may vary significantly. Likewise, chitosan films/coatings can act as a vehicle carrier of bioactive substances such as polyphenolic compounds, bacteriocins, essential oils, or plant extracts to develop novel active packaging materials with antioxidant and antimicrobial properties [9,10]. To improve their antibacterial properties, chitosan edible film also could be combined with different green synthesized materials as silver nanoparticles using plant extracts that help in the reducing, stabilizing, and capping of these nanoparticles [11,12]. However, this technology is taking its first steps and requires many and more in-depth studies to reliably determine all the possibilities it may have.

One of the main advantages of these films is that they allow a slow and controlled migration of the bioactive compounds from the coating to the products. In this sense, several studies have been carried out regarding edible chitosan films embedded with several bioactive compounds in order to evaluate the effects of these substances on the lipid oxidation and microbiological stability of several food products such as meat, fish, and seafood products, dairy products, and fruits and vegetables.

The present review will examine the current literature on both the antibacterial and antioxidant properties of edible films supplemented with bioactive compounds and their applications in food products for their preservation and shelf-life extension.

## 2. Chitosan Edible Films with Added Antimicrobial Bioactive Compounds

In the last years, several efforts have been dedicated to the development of edible films and coatings based on natural bio-materials such as chitosan. As mentioned above, one of the main approaches currently used is the incorporation of bioactive compounds in biodegradable coating bases to provide them with antimicrobial properties. This strategy is regarded as a promising way to develop edible films and coatings with antibacterial or antifungal properties, which could be potentially used as food packaging films. Additionally, natural extracts are considered safe for humans and more environmentally friendly in comparison with the nano-antibacterial agents prepared from different inorganic materials [13]. Among the antimicrobial agents widely incorporated into chitosan edible films are essential oils, plant extracts, or bacteriocins (Table 1). Moreover, it is important to highlight that chitosan is a molecule that presents antibacterial and antifungal activity by itself that could be enhanced by the addition of these substances.

**Figure 3:**
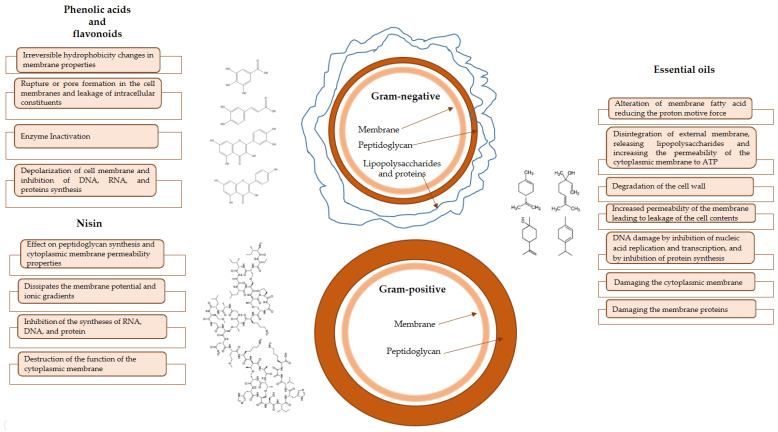
Action mechanism proposed for the different bioactive compounds added to chitosan edible films on microbial Gram-positive and Gram-negative cells.

The mechanism of action has yet need to be completely acknowledged, but the studies carried out so far suggest that this process involves cell lysis, rupture of the cytoplasmic membrane barrier, electrostatic interactions between the positively charged glucosamine monomers of chitosan, and the negatively charged sites of microbial cells, or the chelation of trace metal cations [14]. It is worth noting that the kind of microorganism also plays an important role in comprehending the mechanism behind the antimicrobial activity of chitosan. According to Verlee et al. [15], there are four kinds of microorganisms depending on their susceptibility to chitosan antimicrobial action: Gram-positive bacteria, Gram-negative bacteria, chitosan-sensitive fungi, and chitosan-resistant fungi. Depending on their structure and nature, the antibacterial compounds interact with determined sites of the bacterial cell. Thus, the proposed action mechanisms of the different bioactive compounds added to chitosan films are summarized in Figure 3.

**Table 1:** Antimicrobial compounds added to chitosan-based composites for the development of bioactive chitosan edible films and coatings.

Bioactive Compound	Bioactive Molecules	Concentration	Antimicrobial Effect	Reference
*Eucalyptus* *globulus* EO	Monoterpenes and sesquiterpenes	1.5%	Log reduction of 2.28–4.71 for *Staphylococcus aureus*; 1.85–4.55 for *Bacillus cereus*; 1.81–4.22 for *Escherichia coli* and 1.73–3.98 for *Salmonella enteritis*	[16]
*Thymus* *Piperella* or *Thymus* *moroderi* EOs	Monoterpenes and sesquiterpenes	0.5, 1 and 2%	Inhibition halo of 13.50–19.50 mm for *Serratia marcenscens*; 12.00–19.50 mm for *Aeromonas hydrophila*; 13.50–25.00 mm for *Alcaligenes faecalis;* 12.00–19.00 mm for *Listeria innocua* and 20.00–30.00 mm for *Achromobacter denitrificans*	[17]
*Thymus* *mastichina* EO	Monoterpenes and sesquiterpenes	1 and 2%	Inhibition halo of: 21.15–32.36 mm for *S. marcescens*; 17.92–21.51 mm for *L. innocua*; and 18.42–28.29 mm for *A. faecalis*	[18]
Hop extract	Polyphenolic compounds	0.1, 0.5, 1.0 and 1.5%	Inhibition zones of 1.4–3.0 mm for *Bacillus subtilis*	[19]
Ellagic acid	Polyphenolic compounds	0.5, 1.0, 2.5 and 5.0%	Total inhibition of *S. aureus* and *Pseudomonas aeruginosa* growth	[20]
Maqui Berry	Polyphenolic compounds	0.5 and 1%	Inhibition halo of: 14.65–17.07 mm for *S. marcescens*; 16.82–18.59 mm for *A. hydrophila*; 21.43–22.94 mm for *A. denitrificans*; 20.05–21.03 mm for *A. faecalis*; 12.86–22.03 mm for *Pseudomonas fluorescens*; 17.10–18.67 mm for *Citrobacter freundii* and 14.32–21.89 mm for *Shewanella putrefaciens*	[9]
Tea extracts	Polyphenolic compounds	0.1%	Inhibition halo of 3.7 and 4.8 mm for *S. aureus* and 4.8–9.3 mm for *E. coli*	[21]
Nisin	Bacteriocins	2.5 μg/mL	Inhibition halo of 11.5–12.8 mm for *Listeria monocytogenes*; 10.8–13.3 mm for *B. cereus*; 12.5 mm for *E. coli*; 10.0–12.1 mm for *S. aureus*; 9.5–10.8 mm for *Salmonella enteritidis* and 10.6–11.5 mm for *Clostridium perfringens*	[22]
Nisin	Bacteriocins	Nisin 6000 I.U/mL	*L. monocytogenes*: average inhibition diameter of 15 mm	[23]

### 2.1. Essential Oils (EOs) from Aromatic Plants with Antimicrobial Properties

The rapid emergence of microorganisms resistant to common synthetic antimicrobial compounds has led to the urgent need to find and use alternative compounds to help solve this issue. In this sense, the use of antimicrobial compounds of natural origin such as essential oils has emerged as one of the most promising alternatives due to their antimicrobial properties against a broad range of microorganisms [24]. The use of essential oils is gaining great interest since they are widely accepted by consumers and have been recognized as safe (GRAS) by the US FDA. Essential oils can be used to control or prevent food spoilage caused by the growth of foodborne microorganisms; however, there are several aspects that need to be considered such as the effect on organoleptic properties, range of activity against the target microorganisms of the foods as well as the nutritional composition of the foods [25]. The antimicrobial activity of essential oils is related to the increase in the bacteria cell wall permeability as this results in a dissolution of the lipid structures compromising the integrity of the microbial cell wall, resulting in bacterial cell death. Likewise, EOs can damage membrane proteins and the cytoplasmic membrane, making Gram-positive bacteria more susceptible to their antimicrobial action [26].

The edible films and coatings may reduce the distribution of bioactive compounds with antimicrobial activity into the product since the essential oils form part of the chemical structure of the film and interact with the polymer and the plasticizer [17]. The release of the antimicrobial compounds from the edible films depends on different factors such as electrostatic interactions between the antimicrobial agent and the polymer chains, osmosis, structural changes induced, and environmental conditions [27].

The antibacterial activity of chitosan-based films formulated with essential oils extracted from plants has been extensively studied during the last few years. However, the methodology used to prepare the films, the concentration of essential oil added, the method used to determine the antibacterial activity, as well as the bacterial strains used are very different among the different studies present in the scientific literature, making it very difficult to compare studies.

For instance, Raphaël and Meimandipour [28] carried out a study to assess the antibacterial activity of chitosan film incorporated with thyme and oregano essential oils against different bacterial strains. These authors found that the development of chitosan films with essential oils had a minimum inhibitory concentration of 2.5, 0.625, 2.5, and 5 µL/mL against *Staphylococcus aureus*, *Escherichia coli*, *Enterococcus faecium*, and *Lactobacillus rhamnosus*, respectively, whilst the minimum bactericidal concentration was 5, 1.25, 5, and >10 µL/mL for the same bacterial strains in the same order. The authors stated that the antibacterial activity of the films may be due to the fact that the positively charged chitosan supplemented with essential oils creates a semi-permeable barrier that can reduce respiration and delay microbial growth. Souza et al. [29] analyzed the antibacterial activity of bionanocomposites based on chitosan and sodium montmorillonite embedded with ginger or rosemary essential oil. These authors found that chitosan/sodium montmorillonite bionanocomposite with 2% of ginger essential oil had log reductions of 1.8, 3.8, 3.1, 7.9, and 7.7 against *E. coli*, *Pseudomonas aeruginosa*, *Enterobacter faecalis*, *S. aureus*, and *Listeria monocytogenes*, respectively.

The antibacterial activity against *L. monocytogenes* and *E. coli* of chitosan edible film and chitosan films incorporated with citrus essential oils was assessed by Li et al. [30]. The results showed that the inhibition zone diameters of chitosan against *E. coli* and *L. monocytogenes* were 12.24 and 13.35 mm, respectively, while the inhibition zone diameters of chitosan incorporated with citrus essential oil on *E. coli* and *L. monocytogenes* were 17.23 and 19.19 mm, respectively. These authors also presented the evaluation of the sensory quality of Pacific mackerel treated with chitosan and citrus EO coatings carried out by 37 trained panelists. The results showed that although some odor of the essential oil was detected, this did not affect the sensory acceptability throughout the storage period. In a similar study, Hadidi et al. [31] analyzed the antibacterial activity of clove essential oil encapsulated in chitosan nanoparticles using a two-step technique of emulsion-ionic gelation. These authors reported that chitosan nanoparticles loaded with clove essential oil had an important antibacterial activity against *L. monocytogenes*, *S. aureus*, *Salmonella typhi*, and *E. coli* with inhibition halos of 48.0, 47.8, 44.9, and 39.5 mm, respectively. In the same way, Mohammadi et al. [32] studied the antibacterial activity of biodegradable film developed with chitosan nanofibers and whey protein isolate with added cinnamon essential oil. These authors found that films had inhibition zones of 13.1, 12.3, and 12.2 mm against *E. coli*, *S. aureus*, and *P. aeruginosa*, proving that they represent a promising and suitable option for active packaging.

Recently, Odjo et al. [33] developed an antimicrobial chitosan film using lemon essential oil and cranberry juice. The results showed that these films possessed considerable antifungal activity against *Candida albicans* and *Penicillium roqueforti* with inhibition halos of 11 and 20 mm, respectively. Clove essential oil was encapsulated in halloysite nanotubes and combined with chitosan to develop active films for food [34]. This work showed that the films presented a clear inhibition halo at around 10–11 mm against *E. coli* and *Bacillus mojavensis*, confirming their suitability for application as food-active coatings. It is important to note that this antimicrobial activity is carried out in in vitro studies. The real challenge that must be overcome with the use of chitosan films mixed with essential oils is their application in food. Their use will depend on numerous factors such as the type of food matrix, temperature and storage time, storage conditions, and, most importantly, from our point of view, the possible changes at the sensory level that may appear.

### 2.2. Plants and Vegetal Extracts with Antimicrobial Properties

Plants and other vegetal raw materials represent an unlimited source of novel phytochemicals compounds, which have potential use in the food industry and other applications. Conventionally, the raw extracts obtained from different parts of several plants, including the root, stem, flower, fruit, and twigs, are commonly utilized for prevention or disease treatments [35]. Crude extracts obtained from plants contain in their composition several phytochemicals including polyphenolic compounds (phenolic acid and flavonoids), alkaloids, and tannins, which can exert antibacterial capacity. This antibacterial activity, in some cases, characteristically results from the combination of these secondary bioactive products. In recent years, several investigations have been carried out with the aim of incorporating extracts from plants in active food packaging films or edible films, particularly in biodegradable polysaccharide films in general and in chitosan films in particular [36]. So far, many plant extracts have been evaluated with reference to their effects on antibacterial properties when they are incorporated into packaging films aimed at food protection as with essential oils added to chitosan films. For plant extracts, the discrepancy in the methodology used for the elaboration of the films is even greater. This makes it very difficult to obtain comparable data between the different studies.

In this context, Riaz et al. [37] incorporated apple peel polyphenols into chitosan to develop a novel functional film with antibacterial properties. These authors reported that chitosan-apple peel polyphenol films exhibited concentration-dependent antimicrobial activities against *E. coli*, *B. cereus*, *S. aureus*, and *S. typhimurium* with inhibitory halo zones ranging from 9.76 to 16.12 mm, 11.66 to19.48 mm, 10.86 to 18.11 mm, and 7.13 to 14.47 mm, respectively. This antibacterial activity is mainly related to the bioactive phenolic compounds that could exert physiological changes in the microbial cell membrane and eventually result in bacteria death [38]. In a similar study, the antibacterial activities of chitosan films incorporated with three different methanol extracts of turpentine tree (*Pistacia terebinthus*) were investigated against food-borne pathogens by Kaya et al. [39]. They found that chitosan edible films with the plant extracts had antibacterial effectiveness against *Proteus microbilis*, *Proteus vulgaris*, *P. aeruginosa*, and *E. coli* with inhibition zones between 28.19 and 21.33 mm. The antifungal properties of chitosan coatings with an ethanolic extract from *Santolina chamaecyparissus* were evaluated by Ortiz de Elguea-Culebras et al. [40]. The coatings with the cotton lavender extract showed a stronger antifungal capacity than the control coatings (only chitosan), reducing the growth of *Aspergillus flavus* by more than 50% after 3 days of incubation. *Penicillium verrucosum* was the most susceptible mold to the antifungal action of the coating, which inhibited 67.7% of the growth after 3 days while the control showed no inhibition.

Qin et al. [41] analyzed the antibacterial activity of active and intelligent food packaging films formulated with chitosan with added anthocyanin-rich purple corn extract. These authors found that the developed film exhibited antibacterial activity against *E. coli*, *Salmonella* spp., *S. aureus*, and *L. monocytogenes* with inhibition zones of 5.40, 5.92, 6.57, and 5.48 mm, respectively. Similarly, Koosha and Hamedi [42] investigated the antibacterial potential of chitosan/PVA films containing 1% of black carrot anthocyanins against some Gram-negative and Gram-positive bacterial strains. These authors found that films with an added rich-anthocyanin extract obtained from black carrot had an antibacterial efficiency (%) against *E. coli*, *P. aeruginosa*, and *S. aureus* of 40.00, 35.63, and 44.56, respectively. These last two studies relate the antimicrobial activity of films to the presence of anthocyanins. Numerous antibacterial mechanisms have been suggested for the action of anthocyanins including destabilization of the cytoplasmic membrane, permeabilization of the plasma membrane, inhibition of extracellular enzyme secretion, direct action on cellular metabolism, and prevention of substrate delivery required for bacterial growth [9]. In a later study, Zhang et al. [43] investigated the potential antibacterial properties of films formulated with chitosan and mangosteen (*Garcinia mangostana* L.) rind powder at different concentrations to develop active packaging. They reported that mangosteen-chitosan films had antibacterial activity against *E. coli* (inhibition halos ranged from 7.18 to 8.49 mm), *Salmonella* (inhibition halos ranged from 7.56 to 8.74 mm), *S. aureus* (inhibition halos ranged from 7.76 to 9.10 mm) and *L. monocytogenes* (inhibition halos ranged from 8.42 to 9.48 mm). These authors affirmed that the antimicrobial action of polyphenols is related to an increase in cell membrane permeability, the deformation of cells, and the inhibition of DNA/RNA synthesis.

In a recently published study, Nadira et al. [44] prepared active carboxymethyl chitosan (CMC) films by incorporating different concentrations (5, 10, 15, and 20%) of cashew leaf extract. The results showed that cashew leaf extract enhanced the antimicrobial activity of the pure CMC films, obtaining inhibition values between 24.49 and 71.93 % against *E. coli,* and between 42.99 and 82.19% for *S. aureus*. Likewise, biodegradable films based on passion fruit peel pectin/chitosan with a bioactive extract from *Piper betle* L. leaf proved to be more effective against different bacterial stains (*Bacillus cereus*, *Klebsiella pneumoniae*, *S. aureus*, and *P. aeruginosa*) than the control films. The inhibition halos obtained ranged from 6 to 9.67 mm for *B. cereus*, 6–10.33 mm for *K. pneumoniae*, 8–11 mm against *P. aeruginosa*, and 11–14.67 mm against *S. aureus* [45].

### 2.3. Bacteriocins with Antimicrobial Properties

Bacteriocins are usually defined as peptides or ribosomal proteins synthesized by bacteria that can inhibit or kill other bacteria either from the same species or from other genera [46]. They are generally produced by lactic acid bacteria (LAB), and their GRAS status has attracted great interest from researchers and food industries due to their application as an antimicrobial agent to prevent the development of undesirable microorganisms (spoilage and pathogens) in both fermented and non-fermented foods [47]. Therefore, these substances can have a narrow spectrum by inhibiting bacteria taxonomically close or a wide spectrum by inhibiting a broad variety of bacteria [48]. The advantages of the bacteriocins are that they are thermostable and hypoallergenic, and they can be easily degraded by proteolytic enzymes of the mammalian gastrointestinal tract [49]. The mechanism of action of the bacteriocins is largely known and these peptides and proteins bind to specific or general cell receptors forming pores in the membrane that cause cell permeability and thus bacterial death [50]. Among the bacteriocins, nisin is the main bacteriocin usually incorporated in chitosan films and coatings to improve their antibacterial properties. This bacteriocin is produced by some strains of *Lactococcus lactis* subsp. lactis., and the polypeptide chain contains l-amino acids and the unusual sulfur-amino acids lanthionine and β-methyl-lanthionine. So far, nisin (E234) is the only bacteriocin approved as a food preservative, being recognized as GRAS by the US FDA in 1988 and normally used at maximum concentrations of 12.5 mg/kg in cheeses to prevent the growth of *Clostridium botulinum* [51], a widely known bacteria for causing contamination and spoilage of dairy products.

Pranoto et al. [52] analyzed the antimicrobial activity of chitosan edible film incorporating nisin at various concentrations (51,000, 102,000, 153,000, and 204,000 I.U./g chitosan). This activity was verified against some food-pathogenic bacteria including *E. coli*, *S. aureus*, *S. typhimurium*, *L. monocytogenes*, and *B. cereus*. These authors found that the chitosan-nisin films were not active on *E. coli* and *S. typhimurium*. However, for *S. aureus*, *L. monocytogenes,* and *B. cereus* the inhibition halos ranged between 22.67 and 26.50 mm; 28.67–31.83 mm, and 22.17–22.83 mm, respectively. Hu et al. [53] developed and optimized microcapsules formulated with chitosan and nisin at a 3.8:1 ratio (*w*/*w*) showing antimicrobial activity against *Bacillus subtilis* with an inhibition zone diameter of 19.85 mm. Similarly, antimicrobial bilayer films using chitosan, cellulose, and nisin at 500 and 1000 μg/mL were prepared using the catalyzed sol-gel technique [54]. The results showed that the films exerted antibacterial activity against *L. monocytogenes* with inhibition zones of 23 and 26 mm at concentrations of 500 and 1000 μg/mL, respectively.

In a recently published study, Chen et al. [55] evaluated the antimicrobial activity of three-layer films based on gelatin-chitosan/nisin-corn starch prepared using the layer-by-layer method. Films without nisin did not inhibit *L. monocytogenes* growth while the incorporation of this bacteriocin increased the antimicrobial spectrum of the films, effectively inhibiting the growth of *L. monocytogenes* and *E. coli*.

## 3. Chitosan Edible Films with Added Antioxidant Bioactive Compounds

Reactive oxygen species (ROS) are responsible for oxidation in both food and biological systems. The oxidation process is one of the main causes of chemical deterioration in many food products due to exposure to air, heat, and/or light. One of the most common oxidative processes in foods is lipid peroxidation, which results in the production of undesirable chemical compounds (e.g., aldehydes, ketones, and organic acids) causing a loss of nutritional value and a reduction in the shelf life of lipid-containing foods [56]. Another oxidative process that occurs in foods during ripening, processing, and storage is enzymatic browning. This mainly occurs in fruits and vegetables and implies the enzymatic oxidation of phenolic compounds with the consequent formation of dark pigments [57].

In biological organisms, ROS causes lipid oxidation in cells, decreasing the membrane fluidity, triggering mutations in cellular DNA and the possibility of cancer [58]. In this context, the incorporation of antioxidants in foods is one of the main approaches used by industries to prevent these oxidative processes.

Antioxidants are substances that can protect cells against oxidative processes due to various mechanisms such as the scavenging species that initiate peroxidation, breaking the auto-oxidative chain reaction initiated by ROS, chelating metal ions to prevent the formation of ROS or decomposing peroxides and decreasing localized oxygen concentrations [59].

Most natural antioxidants are sourced from plants, vegetables, herbs, or spices and can be classified into three groups: phenolic compounds, vitamins, and carotenoids [57]. As mentioned throughout this review, some phenolic compounds, in addition to being the main elements responsible for antioxidant activity, have antibacterial and antifungal activity. In the case of vitamins, the most important are vitamin E, which contains four tocopherols and four tocotrienols, and vitamin C, which can be found naturally present in fruits and vegetables [57]. Likewise, the main carotenoids with antioxidant activity are β-carotene, α-carotene, lycopene, and lutein, the main sources of which are fruits and vegetables [60].

Chitosan, besides its excellent properties as an antibacterial agent, also has antioxidant properties. Thus, in recent years, a wide assessment of the antioxidant and free radical scavenging capacities of chitosan and its derivatives has been reported. As with its antibacterial properties, chitosan has antioxidant activity by itself, and its mechanism of antioxidant activity could be attributed to the residual amino groups of chitosan that form a stable fluorosphere with volatile aldehydes derived from the breakdown of fats during oxidation such as malondialdehyde [61].

On the other hand, it is a common strategy to incorporate bioactive compounds that have demonstrated their antioxidant activity such as essential oils or vegetal extracts in order to improve the antioxidant properties of chitosan films or coatings (Table 2).

### 3.1. Essential Oils with Antioxidant Properties

Essential oils extracted from herbs and spices are an excellent source of phenolic compounds that possess antioxidant properties and have been used for decades as a flavoring agent [69,70]. However, their incorporation in food products has some disadvantages such as lower antioxidant activity compared to synthetics and a potent taste and odor [71]. In this sense, Valdivieso-Ugarte et al. [72] carried out an exhaustive review of the in vitro activity of essential oils and their individual isolated compounds. The results of this study reported that the antioxidant activity of the EOs or isolated compounds from about 50 herbs and spices was evaluated with several methods such as DPPH scavenging activity, BCBT, ABTS, FRAP, and TEAC, observing that the majority of analyzed compounds had a remarkable antioxidant activity, being even more effective than reference antioxidants such as Trolox or ascorbic acid in certain cases.

There are several studies related to the mechanisms of action involved in the antioxidant activity of essential oil compounds, which include free radical scavenging, chain initiation prevention, reducing agents, peroxide termination, singlet oxygen quenching, and metal ion catalyst binding [70,73,74,75].

In this regard, the antioxidant capacity of *Gelidium corneum*-chitosan composite films supplemented with citronella java essential oil was evaluated by Go and Song [76]. The radical scavenging activities of the films increased with the EO concentration reaching ABTS and DPPH values of 43.08% and 51.98%, respectively, at 1.5% java citronella EO. Similarly, Tügen et al. [77] developed gelatin/chitosan films enriched with different concentrations of lemon essential oil (0.25, 0.50, 0.75, and 1%) and observed that the films containing EO displayed antioxidant activity with DPPH values ranging from 13.12 to 60.20%, ABTS 21.43–73.70%, and metal chelating activity from 52.04% to 79.99% while the control films showed no antioxidant capacity.

Nanoencapsulation of bioactive compounds has been proven to be a reliable and effective technique to increase the chemical stability of the compounds by protecting them from volatilization and extending their effectiveness [78]. In this sense, Su et al. [79] encapsulated cinnamon essential oil (0.1–0.38%) in chitosan nanoparticles by oil-in-water emulsification and ionic gelation to develop novel biodegradable films.

This study showed that the incorporation of the essential oil into the films resulted in increased antioxidant activity (DPPH radical scavenging values ranging from 53.4% to 56.9%), and the authors suggest that it may be due to a synergistic effect between the phenolic compounds of the EO and chitosan.

In a subsequent study carried out by Cazón et al. [80], biodegradable films were developed from chitosan and tea tree essential oil at two concentrations (0.5 and 1%). The incorporation of the EO in the films substantially increased the antioxidant activity measured as DPPH and ABTS scavenging activities, and the authors stated that this is ascribed to the terpenic compounds of the essential oil. Likewise, ginger essential oil nanoemulsion at different concentrations (0.5%, 1.0%, 1.5%, 2.0%, and 3.0%) was incorporated into a blend of chitosan and fish sarcoplasmic protein films to evaluate its antioxidant capacity [81]. The results showed a significant increase in DPPH radical scavenging activity, reaching values higher than 80% in the films formulated with the ginger EO nanomenulsion. The authors explain that this increase may be related to a synergistic effect between various compounds of the essential oil rather than to a particular individual compound.

Recently, edible films were formulated with chitosan, casein, and oregano EO (0.5%, 1%, and 1.5% *v*/*v*), showing a significant increase in DPPH radical scavenging with values of 38.03%, 50.98, and 70.40% for the 0.5%, 1%, and 1.5% *v*/*v* of oregano EO, respectively, while the control film (chitosan/casein) had a value of 17.32% [82]. The authors relate the potent antioxidant activity of oregano essential oil to its phenolic composition and terpenoids such as carvacrol and thymol.

### 3.2. Plants and Vegetal Extracts with Antioxidant Properties

Antioxidant compounds are classified as either synthetic or natural origin, with butylated hydroxyanisole (BHA) and BHT butylated hydroxytoluene the most commonly used synthetic antioxidants [83]. However, the growing concern among consumers about synthetic antioxidants and their possible link to health problems has led researchers and food industries to collaborate closely in the development of safer natural antioxidants from natural sources [84].

In this context, vegetal and plant extracts obtained from several sources such as fruits (grapes, pomegranate, date), plants, or vegetables (broccoli, potato, pumpkin) have been widely studied for their antioxidant properties and their ability to reduce or prevent lipid oxidation [83]. Most of these extracts do not have a unique composition of phenolic compounds, being a mixture of various compounds such as carotenoids, anthocyanins, or tocopherols [84,85].

It is also noteworthy to mention that the by-products generated during the processing of these foods and raw materials are also an excellent source of valuable antioxidant compounds whose use allows a reevaluation of their role in reducing the amount of waste generated, and economic losses as well as promoting the circular economy [86,87].

The profile and the concentration of phenolic compounds extracted from the plant and vegetal matrices can vary depending on several factors such as climate, species, cultivation method, and especially the extraction method and solvent used [85]. Regarding the methods used for the assessment of the antioxidant capacity of these extracts, the most commonly used are the DPPH radical scavenging activity, hydrogen radical scavenging assay, hydrogen peroxide scavenging activity, ABTS radical scavenging activity, and reducing power [88].

Powder extracts from pine nut shells, peanut shells, and jujube leaves were incorporated at 0.8% into chitosan films in order to study their antioxidant properties. These plant extracts improved the antioxidant capacity of the films reaching values up to 3.8 times higher than films formulated with only chitosan when winter jujube leaf extract was incorporated (DPPH value of 43.50%). The authors relate this antioxidant capacity to the polyphenol and flavonoid content of the extracts and suggest that the developed films could be useful to inhibit browning and extend the shelf life of foods [36]. Similarly, Rachtanapun et al. [89] evaluate whether the addition of a curcumin extract (0.08, 0.16, 0.24, 0.32, 0.40 mg/mL) improved the antioxidant properties of chitosan films. Chitosan films without the extract showed a scavenging value of 0.71% while the incorporation of curcumin extract at 0.4 mg/mL resulted in a significant increase with a value of 56.82%. These results confirm that the extracts improved the antioxidant capacity mainly due to their phenolic compounds. Pomegranate peel produced after its juice extraction is an excellent source of bioactive compounds with antioxidant capacity such as ellagic acid, punicalagin, quercetin, punicalin, luteolin, kaempferol, glycosides, and pedunculagin [90]. For this reason, the interest in utilizing it as a natural antioxidant has increased in recent years and such is the case of the study published by Kumar et al. [90] in which pomegranate peel extract was incorporated into chitosan films at different concentrations (0.2 g/mL, 0.4 g/mL, 0.6 g/mL, 0.8 g/mL, and 1.0 g/mL). It was noticed that both phenolic content (5.75–32.41 mg/g) and the antioxidant capacity expressed as a percentage of DPPH radical scavenging activity (23.13–76.54%) increased significantly with the concentration of the extract in the films.

Active food films based on chitosan and supplemented with *Artemisia campestris* hydroalcoholic extract and aqueous extract were developed by Moalla et al. [91] in order to evaluate their antioxidant properties. Films were prepared by the casting method and the extracts were incorporated at 1% (*w*/*v*), showing an enhancement in the antioxidant capacity compared to the control films. The films formulated only with chitosan presented a slight antioxidant capacity (10.71%, 6.38%, and 0.132% for DPPH radical scavenging activity, chelating activity, and reducing power, respectively) while those containing the hydroalcoholic extract exhibited the highest values of DPPH radical scavenging activity, chelating effect and reducing power (96.79%, 54.31%, and 0.272, respectively).

Recently, Kahya et al. [92] conducted a study about the incorporation of aqueous extracts from sage and rosemary in chitosan films as a potential food coating with antioxidant properties. The films supplemented with the extracts showed a significant increase in the ability to reduce DPPH radicals (ranging from 10.53% to 84.46%), and FRAP values varied from 3.78 to 25.76 FeSO4.7H_2_O μmol eq/g dry film. Likewise, the authors observed that sage aqueous extract compounds exerted better DPPH activity on the films than rosemary extract, demonstrating that these bioactive films would be effective in preventing oxidative processes in food.

The casting method was utilized to develop a novel green bioactive composite film with antioxidant properties by combining peony leaf extract (0.1, 0.3, 0.5, and 0.7%) and chitosan. The antioxidant capacity of the formulated films increased significantly with the incorporation of peony leaf extract being almost three times higher than the chitosan film. It has been demonstrated that this increase is related to the phenolic compounds of the peony leaf extract, which possess free radical scavenging activity and singlet oxygen quenching ability [93].

## 4. Application of Chitosan Edible Films and Coatings in Foods

### 4.1. Meat Products

The degradation process of meat and meat products can occur basically for three reasons: (i) lipid oxidation and (ii) spoilage caused by microorganisms and (iii) enzymatic action. Lipid oxidation can be subject to several factors such as fatty acid profile, the occurrence of pro-oxidant substances such as certain transition metals, and the presence and concentration of natural antioxidants such as vitamin E. The oxidation products may cause rancid odors and flavors, decrease the shelf life, alter texture and color, and decrease the nutritional values of products due to the degradation of proteins, carbohydrates, and vitamins [94]. The microbial population may come from the animal during the slaughter and gutting process or may occur whilst the meat is treated, cut, packed, transported, retailed, or handled. Bacterial or fungal growth in meat and meat products may result in slime development, structural component degradation, reduction in techno-functional properties such as water holding or emulsion, changes in texture and color as well as off-odors. Enzymatic degradation of carbohydrates, fats, and proteins of the muscle provokes softening and greenish discoloration of meat leading to microbial spoilage [95]. The growing concern of consumers about the presence of synthetic preservatives and preference for natural additives along with the contamination problem caused by the excessive use of plastics in the food industry has promoted the search for more natural, renewable, and environmentally friendly alternatives. Of particular interest are the films and coatings developed from chitosan and incorporated with bioactive substances that prevent spoilage, contamination, or deterioration of meat and meat products, extending their shelf life and fulfilling consumer demands (Table 3).

The shelf life of fresh beef was improved by the application of coatings developed from chitosan/pomegranate peel extract and supplemented with *Thymus kotschyanus* EO (0.5, 1.0, and 2.0% *w*/*w*) over 21 days of storage at 4 °C [106]. The results of the study showed that the coatings effectively inhibited bacterial counts and lipid oxidation, reaching a TBARs value reduction of around 50% with respect to the uncoated samples. Similarly, Zhang et al. [107] developed coatings based on chitosan and gelatin incorporated with free or nanoencapsulated tarragon essential oil to extend the shelf life of pork slices during 16 days of refrigerated storage. The results showed that the coatings could reduce the deterioration of the pork slice quality given that TVC values remained below the threshold established as acceptable (7 log CFU/g) and TBARs values below 1.5 mg MDA/kg at the end of the storage period. The authors were able to extend the pork slice shelf life by 8 and 12 days with the coatings formulated with free and nanoencapsulated tarragon essential oil, respectively.

Langroodi et al. [108] also reported a reduction in mold-yeast and *Enterobacteriaceae* counts when they applied chitosan coatings enriched with oregano essential oil (1% *v*/*v*) and grape seed extract (1 or 2% *v*/*v*) to skinned turkey breast fillet during 20 days storage. The results showed that these coatings were also able to reduce lipid oxidation with significantly lower TBARs values than the control samples, reaching values between 1.16 and 1.29 mg MDA/kg vs. 1.56 mg MDA/kg at the end of storage. Antioxidant and antimicrobial properties of chitosan coatings containing *Artemisia fragrans* essential oil (500, 1000, and 1500 ppm) were demonstrated on the chicken breasts during refrigerated storage with the aim of extending their shelf life [109]. The study showed that these coatings were able to reduce TVC values (from 8.01 to 5.32 log CFU/g), coliform counts (8.58 vs. 3.87 log CFU/g), and mold-yeasts (from 7.55 to 4.27 log CFU/g) when the highest concentration of essential oil was incorporated. Likewise, essential oil incorporation significantly reduced TBARs (1.92 vs. 1.61 mg MDA/kg) and total volatile nitrogen (TVB-N) values from 151.2 to 25.3 mg/100 g. These results indicate that chitosan bioactive coatings with 1500 ppm *Artemisia fragrans* EO were capable of prolonging chicken breast stability and are presented as a potential alternative to commercial plastic-based coatings.

Later, the efficacy of chitosan films supplemented with oregano and thyme essential oils (added at concentrations of 0.5 and 1.0% *w*/*v*) against spoilage and pathogenic bacteria affecting meat was investigated by Gaba et al. [110]. The developed films were able to reduce psychrophilic bacteria growth from 7 to 1.2–2.3 log CFU/g and LAB from 6.9 to 1.5–1.7 log CFU/g in up to 30 days of storage. In addition, the growth of *Pseudomonas* was reduced to 0.6–1 log CFU/g compared to the control (6 log CFU/g) with films containing oregano essential oil. Regarding the growth of pathogenic bacteria, the study showed that the control films were not able to inhibit them, while those formulated with essential oils showed a reduction of ~2.3 and 1 log unit of *E. coli* and 3–4 log CFU/g in the case of *S. aureus*, remaining constant until the end of the study period. Based on the results, these authors were able to extend the beef shelf life by ~10 days compared to samples coated only with chitosan, and those coated with chitosan combined with essential oils also showed enhanced sensory acceptability for up to 25 days, confirming the suitability of the films for improving the quality and safety of fresh or refrigerated meat products. Pérez-Córdoba et al. [111] used gelatin:chitosan-based films incorporated with nanoencapsulated garlic essential oil/α-tocopherol (0.4% *v*/*v*) to evaluate their effect on the shelf life of sliced omega-3 rich mortadella stored for 7 days at 6 °C. Their antimicrobial capacity against pathogenic bacteria was evaluated by inoculating the mortadella slices with *L. monocytogenes* and *P. aeruginosa*, and a significant reduction was observed in the counts of the coated samples compared to the control. This reduction was also observed in aerobic mesophilic and psychrotrophic (between two and three logarithmic cycles) bacteria, and LAB proved to be the most sensitive to the effect of the bioactive coating since no growth was detected in the coated mortadella slices. In addition, the active films were able to maintain the TBARs values below 1.0 mg MDA/kg for up to 5 days of storage, and it can be deduced that the lipid oxidation of omega-3-rich mortadella slices can be delayed, thus extending their shelf life.

It is important to note that these studies, in general, do not take into account the effects that these types of bioactive compound, mainly essential oils, exert on the organoleptic properties of foods. In general, these studies only analyze the effect on the increase in shelf life without considering the opinion of consumers on the final product. It would be convenient, from our point of view, to include in these studies various analyses of sensory acceptance and purchasing intention.

### 4.2. Fish and Seafood Products

Fish and seafood products are highly perishable foods due to their high water content, which provides good conditions for microbial and biochemical spoilage reducing their shelf life. Fish and seafood spoilage can be categorized according to three mechanisms: (i) autolytic spoilage, (ii) oxidative spoilage, and (iii) microbial spoilage. Protein degeneration by native enzymes begins just after the end of rigor mortis and in combination with the production of biogenic amines, the products of proteolysis can serve as nutrients for microbial growth leading to spoilage [112]. In the case of oxidative spoilage, lipid oxidation leads to various problems such as the development of off-flavors, changes in texture and color, and alterations in nutritional value such as loss of fat-soluble vitamins [113,114]. Finally, microbial spoilage is the main factor responsible for the loss of quality of fish, accounting for about 25–30% of the losses in the sector [112].

Therefore, it is very important to adopt adequate preservation methods to maintain the quality and prolong the shelf life of these products. The most commonly used preservation methods in the fish processing industry are the traditional chilling, freezing, salting, and drying approaches along with modified atmosphere packaging (MAP), vacuum packaging, irradiation, and high-pressure processing [115]. However, these preservation techniques are not sufficient to completely inhibit microbial growth and lipid oxidation, and the addition of synthetic preservatives (sodium benzoates, sodium nitrite, BHA, and BHT) is also one of the main approaches employed by the industry. In this regard, natural preservatives such as essential oils and other plant extracts in combination with chitosan have emerged as potential substitutes with their broad antimicrobial spectrum and excellent antioxidant properties [112] as shown in Table 4.

In this regard, the effect of chitosan coatings formulated with lemon verbena extract and essential oil on the shelf life of vacuum rainbow trout (*Oncorhynchus mykiss*) was investigated by Rezaeifar et al. [125]. The results of the study showed that the treated samples had lower values of TBRAs, peroxide values, and total volatile basic nitrogen as well as lower levels of H_2_S-producing bacteria. The sensory evaluation performed by ten trained panelists revealed that the incorporation of lemon verbena EO improved the sensory quality of the fish at the end of storage. Ehsani et al. [126] conducted a study on the use of chitosan films incorporated with sage essential oil on fish burgers of common carp meat to control spoilage caused by bacteria. The results showed that the coatings effectively inhibited the growth of spoilage and pathogenic bacteria at the end of the storage period compared to the uncoated samples (from 8.49 to 5.53 log CFU/g and *Pseudomonas* spp. from 7.82 to 5.12 log CFU/g). Moreover, counts of *Shewanella* spp., considered one of the main fish spoilage bacteria due to its characteristics (psychrotolerant, aerobic and anaerobic growth, and tolerance of a wide pH range), were also analyzed, obtaining a reduction from 7.29 to 5.64 log CFU/g after 20 days of storage. The incorporation of sage essential oil prevented the samples from producing off-odors, obtaining overall sensory acceptability while the control samples were unacceptable at the end of the storage period.

Lipid oxidation results in the formation of potentially toxic compounds that cause fish degradation and unpleasant flavors. Therefore, Demircan et al. [127] applied chitosan coatings enriched with lemon essential oil (1% *v*/*v*) and ethyl lauroyl arginate (0.1% *w*/*w*) to mackerel fillet, noticing an increased antioxidant capacity compared to untreated samples (7.94 vs. 3.75% DPPH radical scavenging) at the end of the storage period. Smart films based on chitosan/Methylcellulose incorporated with anthocyanin from *Phyllanthus reticulatus* ripened fruit (5 and 10 wt%) were applied to fish fillets to monitor their freshness. The developed films changed color upon pH variations (caused by the formation of volatile nitrogen compounds after protein degradation), with a change from dark gray (fresh) to light yellow when spoilage occurred in the fillets [128].

A current study carried out by Zamani et al. [129] revealed that a coating formulated with a blend of chitosan and an aqueous extract of green cumin (added at 1 and 2%) displayed antimicrobial and antioxidant properties to maintain the quality of rainbow trout (*Oncorhynchus mykiss*) fillets. Samples coated with the formulation containing the 2% showed the lowest values of aerobic mesophilic bacteria (3.05 Log CFU/g) and lipid oxidation (0.506 mEq O_2_/kg and 1.53 mg MDA/kg) at 21 days of storage. Recently, Surendhiran et al. [130] performed a modification of a chitosan film using magnetic-silica nanocomposite to encapsulate turmeric essential oil in order to apply it on surimi to extend its shelf life. The films without the nanoparticles effectively inhibited the growth of *B. cereus* for up to 6 days of storage (2.42 log CFU/g), but after that time bacterial multiplication was observed, reaching values of 4.66 log CFU/g at the end of the study time. The authors attribute this fact to an uncontrolled release of the essential oil and showed that this problem was overcome by applying the bionanocomposite film since it exhibited a slower, controlled release of the essential oil, reducing bacterial growth (2.78 log CFU/g at the end of the storage period) and extending the shelf life of the surimi.

### 4.3. Dairy Products

Milk and dairy products such as cheese are an essential part of the daily diet as they contain essential nutrients such as proteins, lipids, minerals, and unsaturated fatty acids. Films and coatings are mainly applied to cheeses to prevent loss of quality during the ripening period, transportation, and commercialization, as these products are particularly prone to microbial spoilage due to their pH, water activity, and nutritional composition [131]. Dipping, brushing, spraying, and electrostatic brushing are the main methods used to apply the coatings in cheese along with the casting film-forming method. The film or coating creates a protective barrier between the food and the surrounding environment, extending its shelf life [132]. The packaging materials used so far by the dairy industry are petroleum-based, non-biodegradable, and not fully recyclable, which currently represents a serious contamination issue. This concern has led the industry to search for alternative biodegradable materials that preserve the quality and safety of dairy products while extending their shelf life. In this way, natural lipids, waxes, and biopolymers such as chitosan are currently being used as packaging materials because of their biodegradability, recyclability, edibility, and sustainability [133,134]. In addition, edible coatings can act as carriers of antimicrobial and antioxidant substances to delay or prevent the development of microorganisms and lipid oxidation in cheese [131], resulting in a product with high safety, quality, and shelf life.

After carrying out a comprehensive review, it has been observed that the literature available so far is more limited in comparison with the numerous studies that have already been done on the application of chitosan films enriched with natural bioactive compounds in food products, so there is a clear need for further research in this field (Table 5).

A chitosan/guar gum/zinc oxide bionanocomposite coating enriched with roselle calyx extract was applied to Ras cheese to evaluate its properties during ripening in comparison with uncoated cheese [138]. The results of the study showed that the coating formulated with 3% roselle calyx extract/zinc oxide was able to drastically reduce mold and yeast counts (4.58 vs. 1.70 log CFU/g) and psychotropic bacteria from 3.65 to 1.08 log CFU/g at 4 months of ripening compared to the uncoated cheese. Similarly, Ríos de Benito et al. [139] reinforced an edible coating based on sodium alginate/chitosan and oregano essential oil with silica particles for its application to Panela cheese. Coated cheese showed a reduction of 1.75 Log CFU/g of mesophilic aerobic bacteria compared to the untreated sample after 15 days of storage, thus improving the quality and shelf life of Panela cheese.

In a more recent study, the effect of chitosan/gelatin coating reinforced with papaya leaves and thyme extract on the quality and shelf life of Kareish cheese during chilled storage was evaluated by Hassan et al. [140]. The extracts were incorporated into the chitosan-gelatin blend coating base at 2%, and a significant reduction in total mesophilic aerobes compared to untreated samples (from 5.70 to 3.76–4.99 log CFU/g) was observed at 24 days of the storage period. Likewise, *Enterobacteriaceae* counts reached 4.45 and 6.39 log CFU/g for films with thyme extract and those with papaya leaves, respectively, while the control samples displayed a value of 8.55 log CFU/g at the same time of ripening. The lowest count of yeasts and molds was recorded in coated samples at 24 days of storage compared to the untreated cheese (8.87 vs. 3.42–4.56 log CFU/g).

### 4.4. Fruits and Vegetables

Nowadays, consumers are more concerned about their health and about spending as little time as possible on the preparation of meals. In this context, fruits and vegetables are associated with a healthy diet in addition to meeting these demands for ready-to-eat products. These foods are rich in vitamins, essential minerals, antioxidant compounds such as polyphenols or flavonoids, and dietary fiber, and their consumption can help prevent the development of health problems such as chronic diseases, osteoporosis, or neurodegenerative diseases [141]. Fruits and vegetables are subjected to several adverse conditions during processing and storage, which leads to the loss of their nutritional value, and about 30% of the fruits and vegetables are affected by microorganisms and insects during harvesting or shipping causing lesions of tissues and compromising the integrity of the product. Therefore, these foods are more prone to contamination, enzymatic browning, or unwanted volatile formation along with alteration in their texture [141].

**Figure 4:**
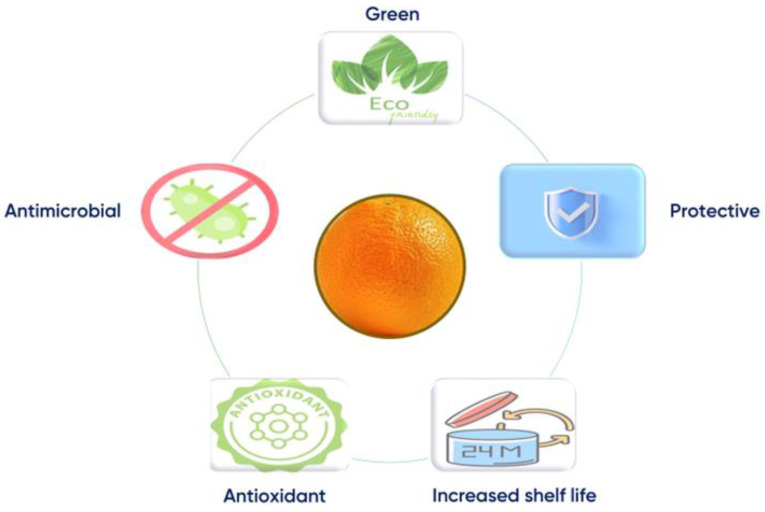
Main beneficial characteristics of chitosan edible films and coatings applied to fruits and other types of foods.

Furthermore, the preservation of minimally processed and ready-to-eat products demanded by consumers is becoming a major challenge as these foods experience rapid deterioration due to an increase in the respiration rate and ethylene production as well as an accelerated consumption of sugars, lipids, and organic acids during the ripening process. All of these modifications can lead to loss of texture and water along with undesirable changes in flavor and color produced by the senescence [142]. These spoilage problems can be prevented by applying edible films or coatings that create a semipermeable barrier to gas and water vapor, keeping the food quality in addition to improving its appearance, flavor, color, and nutritional value (Figure 4).

The aim of the use of coatings and films on fruits and vegetables is to delay the transfer of gas, water vapor, and volatiles resulting in a decrease in respiration, senescence, and loss of aroma, thus retaining moisture and delaying drastic color changes during ripening and storage [142]. In the latter years, it has been widely demonstrated that bioactive chitosan coatings are capable of extending the shelf life of fruits and vegetables by reducing respiration rates, inhibiting microbial development, and delaying ripening (Table 6).

It has been shown that chitosan-nisin films reinforced with silicon dioxide nanoparticles were effective for fresh blueberry preservation at room temperature [150]. The results of the study demonstrated that the incorporation of nisin into the coatings helped to control shrinking (38.52%) and decay rates (8.61%) as well as inhibit the microbial populations for molds/yeast (3.60 log CFU/g) and mesophilic bacteria (2.73 log CFU/g). These coatings were also able to maintain the vitamin C (7.34 mg/100 g) content and polyphenoloxidase (558.03 U/min g) activity. The rapid oxidation process of some fruits such as apples could lead to a shortened shelf life during their postharvest storage and in this regard, Zhang et al. [151] developed antioxidant chitosan films with banana peel extract for quality preservation of apples. The application of the coatings significantly reduced the respiration rate and weight loss over the storage time, and the formulation containing the extract provided an inhibitory effect on apple firmness reduction and was more suitable for maintaining firmness than the pure chitosan film.

In a later study on the application of chitosan coating on fruits, Yang et al. [152] enriched chitosan coatings with turmeric and green tea extracts to be used on strawberries for postharvest preservation. These authors observed that the coating containing turmeric extract inhibited the proliferation of *Botrytis cinerea* during 7 days of storage and the one formulated with green tea extract prolonged the antioxidant properties of the strawberries from 4 to 8 days at 20 °C.

With regards to the application of these bioactive coatings in the preservation of vegetables, chitosan coatings with cinnamon essential oil have been developed to improve the quality and microbiological safety of fresh-cut potatoes [153]. Samples were treated with chitosan films incorporated with different cinnamon EO concentrations (0.2, 0.4, and 0.6% *v*/*v*), and the ones with the lowest dose effectively inhibited the browning, prevented weight loss, and maintained firmness. In addition, there was a reduction in total plate counts, yeast and mold counts, total coliform counts, lactic acid bacteria count, and *L. monocytogenes* (2.14, 1.92, 0.98, 0.73, and 1.94 log CFU/g, respectively). Roshandel-Hesari et al. [82] investigated the antimicrobial and antioxidant properties of edible films based on chitosan/casein with *Origanum vulgare* L. essential oil and its effect on the quality maintenance of cherry tomatoes. At the end of storage, cherry tomatoes with coatings containing the highest EO concentration (1.5%) displayed lower shrinkage and weight loss, thus preserving the quality and firmness of the cherry tomatoes for 32 days.

## 5. Major Challenges and Future Perspectives of Bioactive Chitosan-Based Coatings and Films

The main source of commercial chitosan is chitin, which is the second most abundant polysaccharide preceded by cellulose. It can be obtained from green algae, the cell walls of fungi, the cuticles of insects and arachnids, and in the exoskeleton of crustaceans; the major chitin source used at the industrial level is the shells from crustaceans (shrimp, prawn, crab, and lobster). The industrial production of chitosan involves different chemical processes such as decalcification, deproteinization, decolorization, and deacetylation. Acidic and alkali treatments are extremely harmful to the environment so biological treatments are presented as an alternative method for chitin extraction and chitosan production in a more sustainable manner. In this sense, lactic acid-producing bacteria and proteases have been used for demineralization and deproteinization, respectively [154]. However, despite the high quality of the final product obtained and being environmentally friendly, these methods require several days of processing and are currently limited to laboratory-scale studies [155].

Normally, chitosan is produced using conventional chemical and enzymatic methods, but in some cases, microwave technology has been employed as an alternative to conventional thermal heating because of its increased reaction rates, shorter reaction times, higher yields, energy savings, and a reduction in side reactions in addition to being highly effective for chitosan depolymerization and production of low molecular weight chitosan. This environmentally friendly method greatly reduces extraction times compared to conventional methods (a few minutes vs. several hours), proving to be a viable method to scale up chitosan production [156].

The volatility of resource availability, the residual presence of allergens and/or contaminants that require costly purification and refining of the final products as well as a low degree of deacetylation might limit the industrial scale-up of chitosan production using chitin obtained from shellfish waste [154]. Therefore, the biological production of chitin and chitosan is being regarded as an alternative process to ensure availability and reduce allergen problems, and algae are one of the main sources of biological chitin and chitosan. This is the case with *Cyclotella* sp., which produces extracellular nanofibers composed of β-chitin that can be extruded from the cell [157]. Likewise, chitosan can also be produced from fungi such as those belonging to the Zygomycetes division, which are capable of producing chitosan from chitin synthesis by chitin synthase and transformation into chitosan by chitin deacetylase [154]. One of the main advantages of this biological extraction method is that the fungal biomass can be obtained by simple fermentation at a low cost and that it is considered to be environmentally friendly since it does not require a demineralization step [158].

The manufacturers and suppliers of chitosan and chitin products can be found worldwide and among them are Primex, selling chitosan for packaging applications under the brand ChitoClear^®^, Norwegian Chitosan, trading chitin and chitosan under the brands NorLife and Kitoflok™, respectively, and G. T.C. Bio Corporation, which produces and sells chitin and chitosan at different grades with prices ranging from 18 to 45 EUR/kg for chitosan and around 20 EUR/kg for chitin (depending on the degree of purity) [159].

As demonstrated throughout the present review, the production of chitosan films and coatings has been extensively studied over the last decades; however, most of them utilized the casting method for their production mainly at the laboratory scale. This method consists of dissolving the polysaccharide in a solvent, which is an acetic acid solution in the case of chitosan, and simultaneous incorporation of the plasticizer followed by pouring the mixture on an inert surface for the evaporation of the solvent and the obtainment of a thin film. Therefore, one of the main challenges in the use of chitosan as a new material for the manufacture of packaging is to scale up this method at the industrial level or to find alternative production methods that can replace this method. The application of plasticizing processes by thermomechanical treatments could be an alternative to the traditional casting for the production of films that would allow the manufacture of these biodegradable films on a large scale [160].

Another of the main limitations of chitosan films is that they have poor mechanical properties, so researchers have focused their interest on the use of other compounds such as gelatin, starch, and alginates to improve the mechanical stability of the films [26].

Throughout this review, we have discussed bioactive compounds that can be incorporated into chitosan to develop coatings and films with antimicrobial and/or antioxidant properties. These compounds have been proven to be useful in extending shelf life and maintaining food quality as well as controlling the growth of molds and pathogenic bacteria. However, further work is needed to fully understand the interactions between chitosan and bioactive compounds to optimize their effectiveness as well as to overcome the challenge of maintaining the antimicrobial/antioxidant properties of essential oils or other plant extracts in the films at the high temperatures used in plastics production processes [154]. Moreover, another major challenge faced by the bioactive chitosan-based packaging material for their transfer to the commercial level is their capacity to maintain their activity for long periods since that effectiveness has so far been demonstrated for short periods of time [161]. Nanotechnology is one of the methods that can help to overcome this problem and extend the effectiveness of the compounds. In recent years, studies have been carried out with several nanomaterials that will help to improve the effectiveness of bioactive chitosan coatings and films, opening new opportunities for the design of scalable hybrid materials over the coming years.

## 6. Conclusions

Considering the wide variety of studies on the development of edible and biodegradable coatings and films, it is clear that the food industries have to cut down on the use of plastic materials as packaging due to their well-known disadvantages and the pollution problems they are causing.

The challenges in food preservation have prompted research in the development and use of films and coatings with types of polymers that serve as carriers of bioactive compounds for industrial application in the manufacture of packaging or films to increase the useful life of different food matrices and find the shortest biodegradability time of each element used for this purpose, in addition to being an ecological alternative to plastic packaging, which causes contamination and takes long periods of time to degrade, in contrast to packaging made with natural polymers such as chitosan, which have shown a minimum degradation time.

In this respect, chitosan has proven to be a suitable biodegradable coating material with great potential to become a food packaging material. This review gathered the studies undertaken in recent years on the development of chitosan films and coatings incorporated with bioactive substances of natural origin to improve the stability of products during storage, inhibit microbial spoilage and lipid oxidation as well as increase the quality and shelf life of the foods to which they are applied.

These films and coatings can protect food products by reducing bacterial counts and deterioration reactions such as lipid oxidation or browning to improve their appearance and acceptability. The number of research studies on the development of chitosan coatings is relatively high, but further research is still needed to be undertaken with the aim of finding new sources of bioactive compounds from natural and renewable sources and new polymer blend combinations to improve the characteristics of chitosan coatings in order to compete commercially with current packaging used in the agri-food industry.

## Figures and Tables

**Table 2:** Natural compounds with antioxidant capacity added to chitosan-based composites to produce bioactive films and coatings.

Bioactive Compound	Bioactive Molecules	Concentration	Antioxidant Effect	Reference
*Eucalyptus globulus* EO	Monoterpenes and sesquiterpenes	1, 2, 3, and 4% *v*/*v*	DPPH scavenging: 23.03–43.62% Nitric oxide radical scavenging: 35.23–70.47% H_2_O_2_ radical scavenging: 27.4-63.15%	[62]
*Thymus* EOs	Monoterpenes and sesquiterpenes	1 and 2%	DPPH increased from 0.01 to 0.31–0.55 mg Trolox Equivalent (TE)/g film Ferric reducing antioxidant power (FRAP) increased from 0.08 to 1.98–4.70 mg TE/g film	[18]
*Ziziphora* *clinopodioides* EO	Monoterpenes and sesquiterpenes	1% *v*/*w*	DPPH scavenging activity: ca. 29%	[63]
Ginger EO	Monoterpenes and sesquiterpenes	0.5 g/g biopolymer	Trolox-equivalent-antioxidant capacity (TEAC): 3.3 mM	[64]
Cinnamon, guarana, rosemary, and boldo-do-chile ethanolic extracts	Polyphenolic compounds	1%	TEAC cinnamon: 0.31–0.32 mg/L TEAC guarana: 1.5–1.6 mg/L TEAC rosemary: 0.27–9 mg/L TEAC boldo-do-chile: 1.5–1.8 mg/L	[65]
Propolis	Polyphenolic Compounds, vitamins, and minerals	2.5, 5, 10, and 20% *w*/*w*	DPPH scavenging activity: ca. 29–65%	[66]
Ethanolic grape extract	Polyphenolic compounds	1%	DPPH scavenging activity: ca. 25%	[63]
Hardleaf oatchestnut starch	Polyphenolic compounds	0.5, 2, and 8 g/100 mL	DPPH radical scavenging: 88.45%	[67]
Methanol extracts of stem, leaf, and seed from *Pistacia terebinthus*	Polyphenolic compounds	1 g	DPPH radical scavenging: 37.21–95.91%	[39]
Mango leaf extract	Polyphenolic compounds and carotenoids	1, 3, and 5 wt%	DPPH radical scavenging: 87.16% FRAP: 3.47 μg ABTS: 8.29 μg GA at 5% of extract	[68]

**Table 3:** Natural extracts added to chitosan-based films and coatings for meat and meat products.

Extract Added	Product	Concentration	Bioactive Molecule	Storage Temperature	Effect	Reference
Thyme EO	Cooked ham	0.0%, 0.5%, 1.0%, and 2.0%	Monoterpenes and sesquiterpenes	3 °C for 6 days	Yeast counts significantly decreased.	[96]
Cinnamon or ginger EOs	Lean pork slices	0.05, 0.20, and 1.00%	Monoterpenes and sesquiterpenes	4 °C for 9 days	Chitosan films inhibited the growth of total microbes and significantly reduced TBARs values at 9 days storage.	[97]
Cinnamon EO	Beef patties	Chitosan Cinnamon EO ratio 1:0.8	Monoterpenes and sesquiterpenes	4 °C for 8 days	*Enterobacteriaceae* counts were 3.23 and 3.86 log CFU/g lower than control.	[98]
*Trachyspermum ammi* EO	Chicken fillets	1.0 and 2.0% *w*/*w*	Monoterpenes and sesquiterpenes	4 °C for 12 days	Reduction in aerobic plate count (from 8.32 to 4.56–4.74 log CFU/g), total psychrophilic count, TPC (from 8.65 to 4.54–4.73 log CFU/g), and coliforms (from 5.62 to 2.14–2.24 CFU/g) at the end of storage	[99]
Peanut skin and pink pepper residue extracts	Restructured chicken product	0.84% and 1.90% *v*/*v*	Polyphenolic compounds	3 °C for 7 days	Films without extracts had a PV value 49% higher. Decreased TBARs values by 50 % (peanut skin) and 64% (pepper residue) compared to control and reduced microbial growth at the end of storage.	[100]
*Satureja khuzestanica Jamzad* EO	Lamb meat	1.0% (*v*/*v*)	Monoterpenes and sesquiterpenes	4 °C for 20 days	Shelf-life extension of 10 days by reduction of microbial growth and TBARS scores below 2.5 mg/kg until day 20 of storage.	[101]
Kombucha tea extract	Minced beef meat	1.0, 2.0, and 3.0% (*w*/*w*)	Polyphenolic compounds	4 °C for 15 days	Extended the shelf life by a reduction in *Staphylococcus* counts from 5.36 to 2.11 log CFU/g in 4 days storage and lower TBARs values than control.	[102]
Anise EO	Chicken burger	0.5, 1.0, 1.5, and 2.0% (*v*/*v*)	Monoterpenes and sesquiterpenes	4 °C for 12 days	Chitosan active films extended shelf life of chicken burgers by reducing TVC, *S. aureus*, TPC, and *P. aeruginosa* values and lipid oxidation with lower levels of TBARs values.	[103]
Rosemary EO	Fresh poultry meat	0.5%, 1.0%, and 2.0% (*v*/*v*)	Monoterpenes and sesquiterpenes	5 °C for 12 days	Improved shelf life by reducing TBARs values from 2.03 to 0.24–0.28 mg MDA/g meat, total mesophilic aerobic bacteria from 10.1 to 8.0–8.1 log CFU/ g meat, and total coliforms from 5.6 to 3.0–4.1 log most probable number (/g meat.	[104]
*Paulownia* *tomentosa* EOs	Ready to cook pork chops	Chitosan:EO of 1:1 (*w*/*w*)	Monoterpenes and sesquiterpenes	4 °C for 16 days	Films kept TVC below the microbiological thresholds after 16 days of storage, decreased LAB counts by 2.15–2.61 log CFU/g, and inhibit lipid oxidation, maintaining TBARs values below 1.25 mg MDA/kg meat at the end of storage.	[105]

**Table 4:** Natural extracts added to chitosan-based films and coatings for fish and seafood products.

Extract Added	Product	Concentration	Bioactive Molecule	Storage Temperature	Effect	Reference
Pomegranate peel extract (PPE)	Pacific white shrimp	1.5%	Polyphenolic compounds	Ice storage for 10 days	The increase in TPC TVB-N values over 10 days of storage was significantly reduced in shrimp treated with the chitosan+PPE coating	[116]
Ethanolic red grape seed extract and *Ziziphora clinopodioides* EO	Minced trout fillet	1.0 and 2.0%	Polyphenolic compounds and monoterpenes and sesquiterpenes	4 °C for 11 days	*Enterobacteriaceae* final population decreased by approximately 1–3 log CFU/g compared to untreated fish samples, and films reduced final LAB count (~2–3 log CFU/g) and *L. monocytogenes* growth	[117]
Pomegranate peel extract	Nile tilapia fillets	0.5, 1.0, 1.5 and 2.0%	Polyphenolic compounds	4 °C for 30 days	Reduction in the increase in *Enterobacteriaceae*, *Salmonella*, *E. coli*, yeast-molds, and *S. aureus* over the 30 days of storage. PV values were reduced from 8.35 to 1.17 mEq O_2_/lipid and TBARs from 0.32 to 0.21 mg MDA/kg at the end of storage.	[118]
Apple extract	Grass carp fillets	0.25, 0.50, 0.7, and 1.0% (*w*/*w*)	Polyphenolic compounds	4 °C for 15 days	Films reduced PV and TBARs values as well as showing an inhibition of the increase in TVB-N values.	[119]
Tomato plant ethanolic extract	Sierra fish fillets	0.3% (*v*/*v*)	Polyphenolic compounds and carotenoids	Ice storage for 15 days	Treatments were capable of delaying the mesophyll counts from 6.98 to 4.81 log CFU/g.	[120]
Lemon or thyme EOs	Grass carp fillets	0.25 and 0.5%	Monoterpenes and sesquiterpenes	2 °C for 16 days	Coatings delayed the increase in TBARs and showed a reduction in TVC from 8.18 to 4.85-5.25 log CFU/g, *Pseudomonas* (7.45 to 5.46-5.67 log CFU/g), *Shewanella putrefaciensa* (from 4.53 to 3.29–3.47 log CFU/g), and Enterobacteria (4.25 *vs*. 3.17–3.39 log CFU/g).	[121]
Propolis extract	*Nemipterus japonicus* fillets	0.1%	Polyphenolic compounds, vitamins, and minerals	4 °C for 12 days	Treatments led to a reduction in total mesophilic count (TMC) and total psychrotrophilic count (TPC), and samples showed no increase in the TBARs values during 12 days of storage.	[122]
Citrus EO	Pacific mackerel	1.5% (*w*/*v*)	Monoterpenes and sesquiterpenes	−3 °C for 12 days	Coatings were able to significantly reduce biogenic amine, TBARs and TVC values.	[30]
Pink pepper residue extract	Salmon fillets	0.6% (*v*/*v*)	Polyphenolic compounds	2 °C for 28 days	Coated samples displayed a reduction in total psychotropic viable count (TPVC) from 7.32 to 5.26 log CFU/g and LAB from 7.05 to 6.28 log CFU/g as well as reduced TBARs values at 28 days of storage.	[123]
Clove EO	Frozen tambaqui fillets	0.08% and 0.16%	Monoterpenes and sesquiterpenes	−18 °C for 120 days	Fillets coated showed the lowest TBARS values almost every time (mean value of 0.61 mg MDA eq/kg). Although consumer panelists were not accustomed to the taste of clove EO in fish fillets, they still found them acceptable.	[124]

**Table 5:** Natural extracts incorporated in chitosan-based films and coatings for dairy products.

Extract added	Product	Bioactive Molecule	Concentration	Effect	Reference
Rosemary and oregano EOs	Goat cheese	Monoterpenes and sesquiterpenes	Chitosan:EO ratio of 1:0.5	Coatings delayed or inhibited *Penicillium* and *Mucor* growth and prevent weight loss.	[135]
Boldo extract	Prato cheese	Polyphenolic compounds	1% (*v*/*v*)	Films exerted protection against oxidation compared to uncoated samples and did not allow psychrotrophic microorganism growth.	[136]
Ethanolic extract from *Santolina chamaecyparissus*	Manchego cheese	Polyphenolic compounds	1% (*w*/*v*)	Coated cheese showed just a few fungal colonies with the untreated samples the most affected by fungal contamination.	[40]
*Mentha aquatica* L. EO	Iranian white cheese	Monoterpenes and sesquiterpenes	0.5, 1.0 and 1.5% (*v*/*v*)	The coating provided a reduction in *S. aureus* (from 4.80 to 0.55 log CFU/g), *L. monocytogenes* (5.89 vs. 0.33 log CFU/g), and *E. coli* was detected with the highest EO concentration.	[137]

**Table 6:** Natural extracts added to chitosan-based films and coatings for fruits and vegetables.

Extract Added	Product	Bioactive Molecule	Concentration	Effect	Reference
Olive leaf and olive pomace extracts	Apple and strawberry	Polyphenolic compounds	10 and 20 g/L	The lowest affected areas by the growth of *Penicillium expansum* and *Rhizopus stolonifera* were 7.33 and 8.00 mm in coated apple and strawberry with CH-OLE 20 g/L, respectively.	[143]
*Lippia sidoides* Cham. essential oil and pomegranate peel extract	Italian tomatoes	Polyphenolic compounds, monoterpenes, and sesquiterpenes	EO: 2.5, 5 and 10 mL/L PPE: 5, 10 and 20 mL/L	Coatings delayed the ripening by lowering weight loss and maintaining constant firmness compared to uncoated samples at 12 days of storage.	[144]
Procyanidins extracted from grape seeds	Fresh blueberries	Polyphenolic compounds	0.8% (*w*/*w*)	Maintained the overall quality of fresh blueberries during 14 days of storage at 4 °C.	[145]
Olive leaves extract	Sweet cherries	Polyphenolic compounds	1%	Weight loss was significant, and the highest antioxidant activity was recorded lower in samples treated with the chitosan coatings.	[146]
*Stevia rebaudiana* extract	Fresh-cut apple slices	Polyphenolic compounds	2.5%	Samples coated with the films containing the extract showed higher polyphenoloxidase (PPO) activity and antioxidant capacity values (12.02 μmol TE/g).	[147]
*Ficus hirta* Vahl. fruits extract	Xinyu Tangerines	Polyphenolic compounds	6.25 g	Edible coating reduced weight loss, respiration rate, and malondialdehyde (MDA) content during storage, and activities of superoxide dismutase (SOD), peroxidase (POD), and phenylalanine ammonia-lyase (PAL) were higher in coated samples.	[148]
*Byrsonima* *Crassifolia* extract	Bell pepper	Polyphenolic compounds	2.5 and 5.0%	Edible coatings reduced the microbiological activity by 85% after 21 days of storage and increased secondary metabolites.	[149]

## Data Availability

The data presented in this study are available on request from the corresponding author.
